# Atom-Driven and Knowledge-Based Hydrolysis Metabolite Assessment for Environmental Organic Chemicals

**DOI:** 10.3390/molecules30020234

**Published:** 2025-01-09

**Authors:** Zhe Liu, Yufan Lin, Qi He, Lingjie Dai, Qinyan Tan, Binyan Jin, Philip W. Lee, Xiaoming Zhang, Li Zhang

**Affiliations:** 1 Innovation Center of Pesticide Research, Department of Applied Chemistry, College of Science, China Agricultural University, Beijing 100193, China; 2 Key Laboratory of National Forestry and Grassland Administration on Pest Chemical Control, China Agricultural University, Beijing 100193, China

**Keywords:** machine learning, expert system, metabolite evaluation, hydrolysis sites, hydrolysis products

## Abstract

The metabolism of environmental organic chemicals often relies on the catalytic action of specific enzymes at the nanoscale, which is critical for assessing their environmental impact, safety, and efficacy. Hydrolysis is one of the primary metabolic and degradation reaction pathways. Traditionally, hydrolysis product identification has relied on experimental methods that are both time-consuming and costly. In this study, machine-learning-based atomic-driven models were constructed to predict the hydrolysis reactions for environmental organic chemicals, including four main hydrolysis sites: N-Hydrolysis, O-Hydrolysis, C-Hydrolysis, and Global-Hydrolysis. These machine learning models were further integrated with a knowledge-based expert system to create a global hydrolysis model, which utilizes predicted hydrolysis site probabilities to prioritize potential hydrolysis products. For an external test set of 75 chemicals, the global hydrolysis site prediction model achieved an accuracy of 93%. Additionally, among 99 experimental hydrolysis products, our model successfully predicted 90, with a hit rate of 90%. This model offers significant potential for identifying hydrolysis metabolites in environmental organic chemicals.

## 1. Introduction

The environmental fate of organic compounds is a key topic in environmental chemistry research [1,2,3]. Hydrolysis, a process where organic chemicals are transformed into new chemical species in aquatic environments, plays a critical role in determining their stability [4]. It is one of the primary pathways for the transformation of organic chemicals in the environment [5]. Hydrolysis reactions can occur through hydrolase metabolism (biotic) and non-enzymatic degradation (abiotic) [6]. The hydrolases in plants can specifically bind to agrochemicals at the nanoscale, catalyzing hydrolysis reactions to reduce their toxicity [7]. Most hydrolytic transformations in the environment focus on the environmental fate of agricultural chemicals [8], as these compounds are widely used in the environment and pose significant ecological risks [9]. Hence, the rapid identification of hydrolysis products, whether from biotic or abiotic processes, is crucial for assessing the environmental fate of organic compounds.

The structure of hydrolysis products can be determined using liquid chromatography/mass spectrometry (LC/MS) or liquid chromatography/tandem mass spectrometry (LC/MS/MS) [10]. However, these techniques depend on extensive biological experiments and costly analytical equipment, making them time intensive and impractical for large-scale evaluations involving thousands of chemicals. Consequently, there is a growing focus on developing reliable computational methods to predict and analyze compound metabolic reactions [11,12].

The site of metabolism (SOM) and metabolite structures are two critical areas of research in computer-aided metabolic prediction, offering valuable support and guidance for experimental studies [13]. Numerous studies have concentrated on predicting SOMs. For example, XenoSite [14] utilizes deep learning models trained on extensive experimental data to predict the metabolic sites of compounds within the CYP450 enzyme system. RS-predictor [15] integrates molecular mechanics with statistical methods to assess metabolic stability and identify potential metabolic sites. These studies, however, primarily focus on CYP450-catalyzed reactions, with limited attention to hydrolysis reactions, and are restricted to identifying unstable sites. To overcome these limitations, knowledge-based methods such as MetabolExpert [16], UM-PPS [17], and SyGMa [18] have been introduced, which use encoded chemical reaction rules to predict and explain the chemical transformation processes. However, this approach typically generates a large number of metabolites, potentially leading to a “combinatorial explosion” problem. Recently, BioTransformer [19], GLORY [20], and GLORYx [21] have combined machine learning with knowledge-based methods to account for the prioritization of metabolic reactions, thereby improving the accuracy and efficiency of the predictions. However, although these models perform well with pharmaceutical organic molecules, their precision drops significantly when applied to agrochemicals [22]. Therefore, it is essential to construct a specified assessment model of the biotic and abiotic hydrolytic metabolism of these compounds to better understand their environmental fate in the open ecosystem.

In this study, we developed three atom-driven independent hydrolysis site prediction models (N-Hydrolysis, O-Hydrolysis, C-Hydrolysis) and one global hydrolysis site prediction model (Global-Hydrolysis) using machine learning methods. These models were integrated with knowledge-based expert systems to provide a robust tool for predicting hydrolytic metabolite products. An external test set was employed to verify the predictive quality of our model, and the strengths and limitations of the model were subjected to detailed analysis. This work presents a valuable computational strategy for the assessment of hydrolytic processes in both environmental and biological systems.

## 2. Results

### 2.1. Dataset Analysis

Three independent datasets were constructed, each dedicated to a single atomic type: N, O, or C (N-Hydrolysis, O-Hydrolysis, and C-Hydrolysis). These datasets collected the hydrolysis reaction data specific to their respective atom types. Due to the limited data for sulfur atoms and halogens, they were included in the global dataset (Global-Hydrolysis) covering all atomic types.

The statistics for the metabolic sites are shown in Table 1. In the Sourcing Data, the N-Hydrolysis dataset contained 201 positive samples (SOM+) and 280 negative samples (SOM−), indicating a relatively balanced ratio between the positive and negative samples. However, for the other datasets, there was a significant imbalance between the number of positive samples (SOM+) and negative samples (SOM−): the ratio of positive to negative was 0.28 for O-Hydrolysis, 0.03 for C-Hydrolysis, and 0.24 for Global-Hydrolysis.

The imbalance in the Ext Data, with only 113 positive samples out of 1690 atoms, reflects the natural selectivity of hydrolysis reactions under chemical and environmental conditions rather than bias from the data collection or preprocessing. This imbalance suggests that training the model on Sourcing Data could reflect the real-world phenomenon where hydrolysis sites are less frequent than non-hydrolysis sites, thereby enabling the model to accurately predict these rare hydrolysis sites when applied to real-world data.

### 2.2. Hydrolysis Site Prediction Models

#### 2.2.1. Evaluation of Pre-Trained Model Performance

After the feature selection using the variance threshold, the ANOVA F-test, and the *t*-test, the N-Hydrolysis, O-Hydrolysis, C-Hydrolysis, and Global-Hydrolysis models retained 51, 101, 51, and 188 features, respectively. These models were then pre-trained using eight different machine learning algorithms. The 10-fold cross-validation average accuracy on the training set for each model is shown in Table 2. Overall, the RF model performed the best across all of the reaction types, especially in C-Hydrolysis and Global-Hydrolysis, with accuracy rates of 0.978 and 0.948, respectively, demonstrating strong predictive capabilities. LDA and ADA showed relatively good performance in Global-Hydrolysis, with accuracy rates around 0.910, indicating their potential for handling complex reactions. In contrast, the QDA model exhibited weaker performance across all of the reaction types, especially in O-Hydrolysis and Global-Hydrolysis.

There was no single model that performed best across all of the reaction types; the different models exhibited strengths in different reaction types. Based on their performance, we selected the top five models for each reaction type for further optimization: N-Hydrolysis (DT, RF, ET, LightGBM, and LDA), O-Hydrolysis (ET, RF, GBC, ADA, and LightGBM), C-Hydrolysis (DT, RF, ET, GBC, and QDA), and Global-Hydrolysis (DT, RF, ET, GBC, and LightGBM).

#### 2.2.2. Model Optimization Results

Hyperparameter tuning was performed on the top five models for each reaction type, with 50 different hyperparameter combinations selected for each model and 10-fold cross-validation conducted for each combination. Ultimately, the optimal hyperparameter set for each model was determined based on the average cross-validation accuracy of the training set. The results, as shown in Table S1, indicate that not all of the models experienced significant optimization. Most of the models retained their default parameters, suggesting that the default parameters already provided the optimal predictive performance in these cases. After optimizing the reaction type parameters, slight improvements were observed in the LightGBM model for N-hydrolysis, as well as in the GBC models for O-hydrolysis, Global-Hydrolysis, and C-hydrolysis, with the accuracy increases ranging from 0.2% to 1.6%. The tuned GBC model demonstrated higher accuracy in the Global-Hydrolysis reactions. This improvement was primarily due to lowering the learning rate from 1.0 to 0.15, which resulted in smaller update steps during the model training, allowing for finer learning of the data patterns and avoiding the underfitting caused by overly rapid convergence. Additionally, increasing the number of decision trees from 100 to 230 enhanced the model’s complexity and stability, improving its ability to capture complex data features. Post-optimization, the accuracy of the models on the training set was very close. For instance, for the O-Hydrolysis reaction, the worst-performing model, ADA, had an accuracy of 0.936, while the best-performing model, RF, had an accuracy of 0.946, with a difference of only 0.01. This minimal difference suggests that training set accuracy alone is insufficient to distinguish the performance of the models. Consequently, each model’s performance on the test set was evaluated to determine the best model for each reaction type.

**Figure 1:**
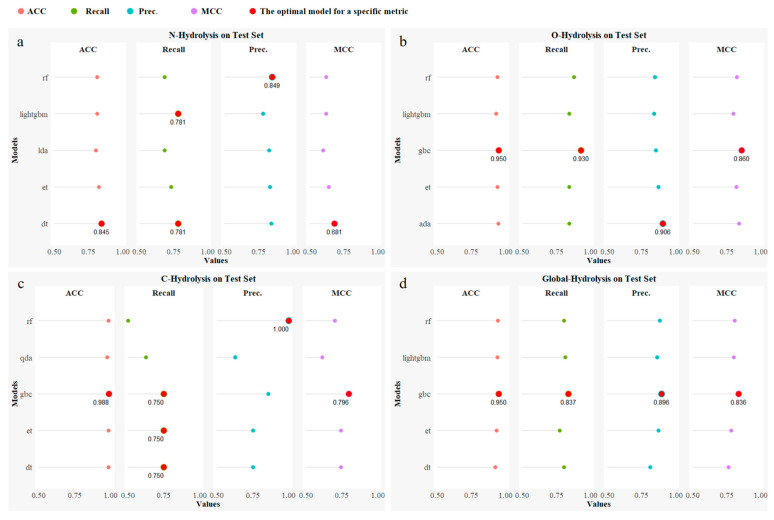
Hydrolysis site prediction model performance on the test set. (**a**) N-Hydrolysis reaction optimized model performance on the test set; (**b**) O-Hydrolysis reaction optimized model performance on the test set; (**c**) C-Hydrolysis reaction optimized model performance on the test set; (**d**) Global-Hydrolysis reaction optimized model performance on the test set. The red circular marks in the figure indicate the model that achieved the best performance for a specific evaluation metric.

The predictive performance of different models on the test set across the four types of hydrolysis reactions (N-Hydrolysis, O-Hydrolysis, C-Hydrolysis, and Global-Hydrolysis) is shown in Figure 1. The results indicate significant variation in model performance depending on the reaction type. For instance, the decision tree (DT) model performed best in the N-Hydrolysis reaction, achieving leading results in accuracy, recall, and Matthews correlation coefficient (MCC), demonstrating its strong predictive capability for this reaction type. In contrast, the gradient boosting classifier (GBC) model excelled in the O-Hydrolysis, C-Hydrolysis, and Global-Hydrolysis reactions, and particularly in the Global-Hydrolysis reaction, where it ranked first in all of the evaluation metrics, showcasing its robustness and generalization ability. This suggests that different hydrolysis reaction types require tailored model selection to optimize their predictive performance.

**Figure 2:**
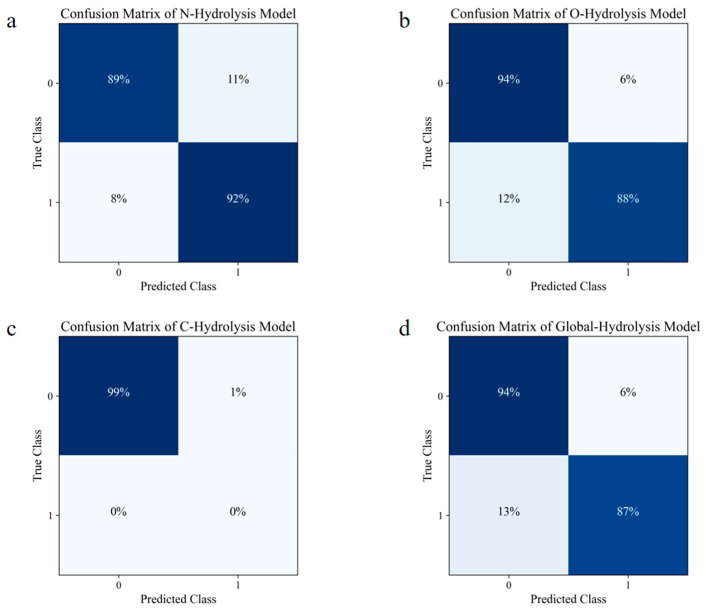
Confusion matrix for each model on the Ext Data. (**a**) Confusion matrix of the N-Hydrolysis model; (**b**) confusion matrix of the O-Hydrolysis model; (**c**) confusion matrix of the C-Hydrolysis model; and (**d**) confusion matrix of the Global-Hydrolysis model.

To further ensure the generalization capability of the models, the best model for each reaction (N-Hydrolysis–DT, O-Hydrolysis–GBC, C-Hydrolysis–GBC, Global-Hydrolysis–GBC) was used to make predictions on the Ext Data. This dataset comprised 75 agrochemical compounds containing 1690 atoms, none of which was used during the model training and optimization. The confusion matrices for each model on the Ext Data are shown in Figure 2. The results demonstrate that these models achieved recognition rates of around 90% for both the positive and negative samples. Notably, the Global-Hydrolysis prediction model successfully identified 94% of the negative samples and 87% of the positive samples. It is important to highlight that for the C-Hydrolysis model, the Ext Data did not contain any positive samples, meaning no metabolizable C atoms were present. Therefore, the model’s prediction for positive samples was 0%, which does not imply the model’s inability to recognize positive samples. Overall, the chosen optimal models exhibited a good predictive performance on the Ext Data, confirming their robustness and reliability in identifying hydrolysis sites in organic compounds.

Additionally, the performance of the Global-Hydrolysis model was compared with MetScore. MetScore is a metabolic site prediction model designed to assess the likelihood of specific atomic sites undergoing metabolic transformations. Among the various metabolic reactions it predicts, hydrolysis reactions are included as a subset. In this study, we specifically compared the performance of the Global-Hydrolysis model with the hydrolysis predictions made by MetScore. However, since MetScore only provided results from cross-validation in the literature, our comparison is based solely on those data. MetScore achieved an average MCC of 0.82 ± 0.02 in 10-fold cross-validation, while our model yielded an average MCC of 0.76 ± 0.04. Although our MCC is slightly lower than that of MetScore [23], it is important to note that MetScore utilized a dataset of 3163 transformations, while we only used 577 transformations—roughly one-fifth the size of MetScore’s dataset. Nevertheless, our model still demonstrated a prediction performance comparable to that of MetScore. Furthermore, unlike MetScore, we conducted external validation on a hydrolysis dataset containing 75 agrochemical compounds, where the atomic information was not included in the training and optimization phases of our model. Our model achieved an MCC value of 0.628 on the Ext Data, indicating that even on unseen data, our model maintains a good predictive capability.

#### 2.2.3. Hydrolysis Reaction Transformation and Evaluation

In this section, the site prediction model and the expert system were combined to achieve accurate predictions of the metabolic products. The site prediction model was first utilized to identify the potential hydrolysis sites within the compounds and generate predicted probabilities for these sites. These prediction results were then fed into the hydrolysis reaction transformation module based on the expert system, which employed the hydrolysis rules from the reaction knowledge base to simulate hydrolysis reactions at these sites, generating corresponding metabolic products. Finally, the generated metabolic products were scored based on their predicted probabilities, ensuring that the products with higher predicted probabilities were prioritized, thus enhancing the accuracy and practicality of the predictions.

The global hydrolysis site prediction model (Global-Hydrolysis–GBC) was applied to the Ext Data to evaluate its performance, as shown in the confusion matrix in Figure 2. The model achieved a prediction accuracy of 87% for the negative samples and correctly identified 94% of the positive samples. The model’s predictions were integrated into the expert system to predict the hydrolysis products for 75 organic compounds in the Ext Data, successfully identifying 90 out of the 99 potential metabolic products (Table 3). Additionally, 13 metabolites that were not observed in the experiments were predicted. These 13 unobserved metabolites may have gone undetected due to their concentrations being below the detection threshold or because they were rapidly metabolized as intermediates. This result demonstrates the effectiveness and potential of our model in enhancing the detection rate of metabolic products and revealing potential metabolic pathways.

To assess the performance of our model, we compared it with GLORYx and SyGMa on the Ext Data. GLORYx identified 98.0% of the products, while both our model and SyGMa identified 91.0%, demonstrating comparable accuracy (Table 3). However, GLORYx and SyGMa do not specify whether the identified products are hydrolysis derived or produced through other metabolic pathways, as these tools primarily focus on phase I and phase II reactions. In contrast, our model is designed to target hydrolysis reactions, providing enhanced specificity in this domain. Importantly, our model achieved a false-positive rate of 12.6%, which was comparable to that of GLORYx (12.6%) and lower than that of SyGMa (15.1%), further underscoring its precision (Table 3). Moreover, our model was built using only 577 parent molecules, significantly fewer than the datasets used by GLORYx (1438) and SyGMa (1848). Despite this smaller training set, our model delivered results on par with both tools, highlighting its effectiveness in hydrolysis prediction.

#### 2.2.4. Application Examples

**Figure 3:**
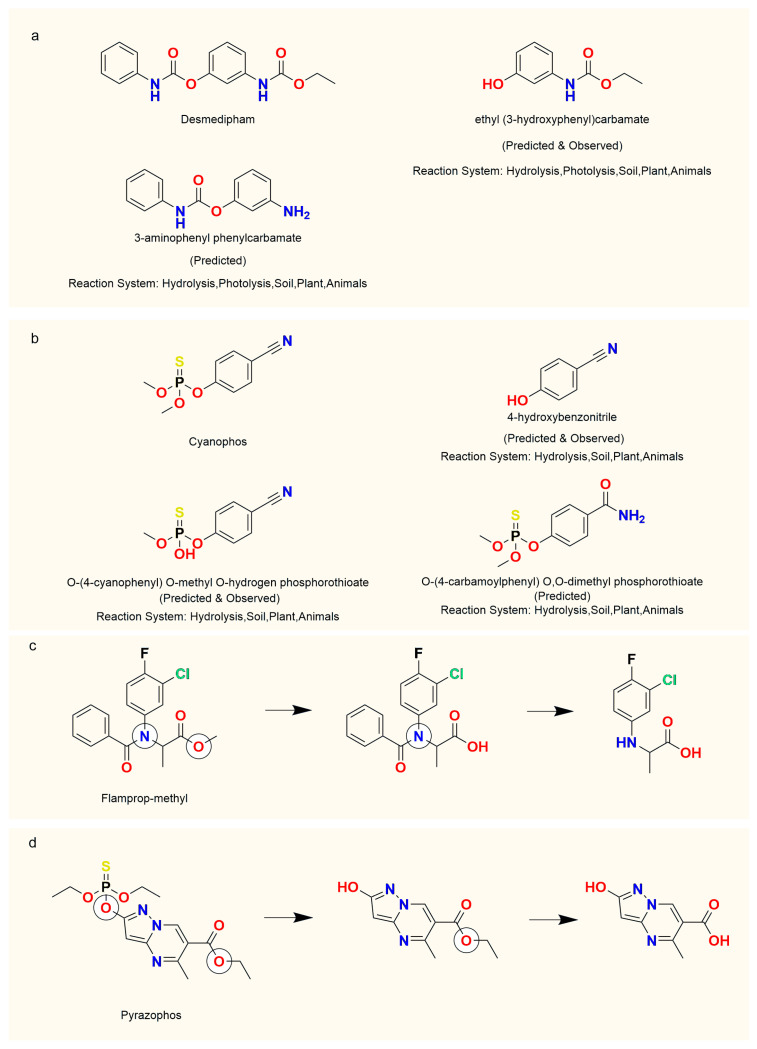
Some examples of metabolites predicted within several molecules. (**a**) Predicted metabolites of Desmedipham; (**b**) Predicted metabolites of Cyanophos; (**c**) Metabolic pathway of Flamprop-methyl; (**d**) Metabolic pathway of Pyrazophos.

Some examples are shown in Figure 3 to demonstrate the performance of the model in predicting and ranking the hydrolysis metabolic products. For instance, the herbicide desmedipham is prone to alkaline-catalyzed hydrolysis (Figure 3a) [24], yielding ethyl (3-hydroxyphenyl)carbamate. Using our model, two potential hydrolysis products, including ethyl (3-hydroxyphenyl)carbamate, were successfully predicted. It was also suggested by our model that hydrolysis of the terminal carbamate of desmedipham would occur, resulting in 3-aminophenyl phenylcarbamate. Another example is the insecticide cyanophos [25] (Figure 3b). In aquatic environments, the alkaline hydrolysis of trialkyl phosphates is typically facilitated by the nucleophilic attack from hydroxide ions on the phosphorus atom, followed by an S_N_2 mechanism to replace the leaving group. This process is also the primary detoxification reaction for organophosphate agrochemicals within insects. Our model successfully predicted three potential hydrolysis products, including three alkyl substitutions on the phosphate ester group and the hydrolysis of the cyano group, which has not been experimentally verified. Furthermore, our model provided insights into the reaction systems where each hydrolysis reaction might occur. These examples illustrate that, in many instances where the predicted metabolites do not fully match the reference metabolites, the predictions still provide valuable information and can guide future experimental validation.

In evaluating the ranking capability of metabolic products, our work also aligns with the product sequences reported in the literature. The herbicide flamprop-methyl (Figure 3c) readily hydrolyzes into the parent acid and methanol at higher pH levels [26]. This is followed by the cleavage of the amide bond, resulting in the formation of C-(3-chloro-4-fluorophenyl) alanine. According to the predictions from the proposed model, the compound flamprop-methyl identified two hydrolysis sites, with the O atom in the ester group scoring 0.9396 and the N atom in the amide group scoring 0.7582. This prioritization of the ester group in the hydrolysis process can be attributed to its higher reactivity and spatial accessibility. The ester bond, characterized by greater electrophilicity and lower bond energy compared to the amide bond, is more susceptible to nucleophilic attack. Moreover, the ester group’s sterically exposed position facilitates easier access for catalytic enzymes or nucleophiles, making it more prone to hydrolysis compared to the amide bond. This reactivity difference, rooted in chemical properties, was incorporated into the model’s predictive rules. Consequently, the predicted metabolic products reflected the preferential hydrolysis of ester groups, aligning with the experimental observations reported in the literature [26].

Similarly, for the fungicide pyrazophos [27] (Figure 3d), the cleavage of the P-O-pyrazole bond is the primary degradation reaction, generating 2-hydroxy-5-methyl-6-ethoxycarbonylpyrazolo [1,5-α]pyrimidine. This is followed by the hydrolysis of the ethyl ester to produce 2-hydroxy-5-methyl-6-carboxypyrazolo [1,5-α]pyrimidine, a step observed only in animals. According to the predictions from the proposed model, the O atom on the P-O-pyrazole bond scored 0.8766, while the O atom in the ethyl ester scored 0.6004, reflecting the metabolic sequence reported in the literature [27]. The selective occurrence of the second step in animals indicates its reliance on specific enzymatic mechanisms or distinct biochemical conditions within the animal metabolism.

## 3. Materials and Methods

### 3.1. Data Collection and Preparation

**Figure 4:**
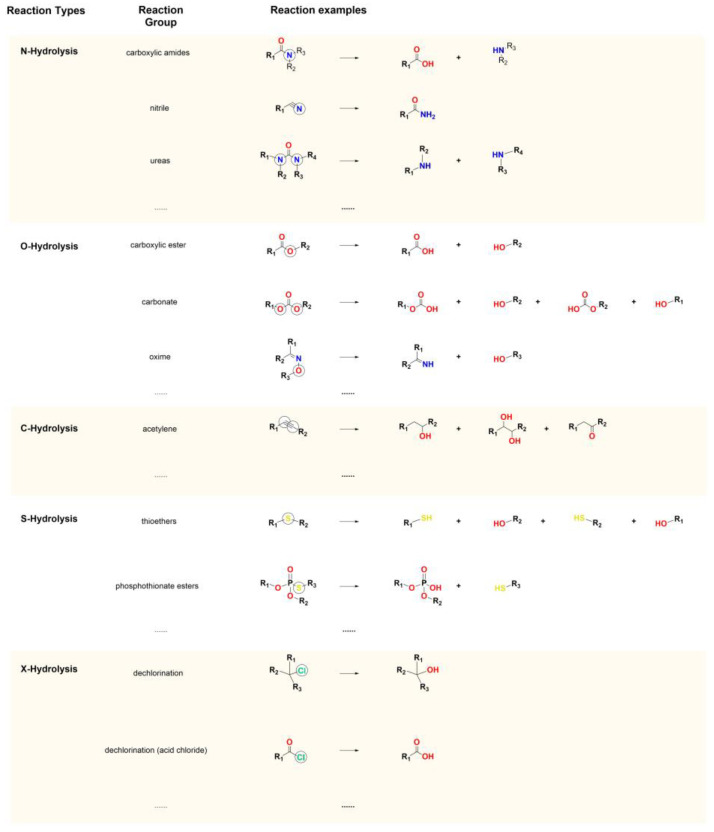
Examples of hydrolysis reactions.

The hydrolysis reactions dataset comprises 577 molecules from Roberts’s Metabolic Pathways of Agrochemicals [25,28], the hydrolysis transformation examples utilized by Tebes-Stevens et al. [8], and the KEGG Reaction Database [29]. By analyzing the chemical structures of the reactants and their hydrolysis products, the hydrolysis sites of the hydrolyzable functional groups were identified (Figure 4). The hydrolysis sites of the nitrogen-containing functional groups, such as carboxylic amides, nitriles, and ureas, were defined as N-Hydrolysis; the hydrolysis sites of the oxygen-containing functional groups, such as carboxylic esters, carbonates, and oximes, were defined as O-Hydrolysis; the hydrolysis sites of the carbon-containing functional groups, such as acetylene, were defined as C-Hydrolysis; the hydrolysis sites of the sulfur-containing functional groups, such as thioethers and phosphothionate esters, were defined as S-Hydrolysis; and the hydrolysis sites of the halogen-containing reactions, such as dechlorination and acid chloride hydrolysis, were categorized as X-Hydrolysis. For each atom type, a corresponding dataset was generated. For instance, the N-Hydrolysis dataset comprised only nitrogen atoms. Within this dataset, the nitrogen atoms at the hydrolysis sites were labeled as positive samples (“SOM+”), while the other nitrogen atoms were labeled as negative samples (“SOM−”). Similarly, distinct datasets were created for oxygen (O), carbon (C), sulfur (S), and halogens. Each dataset was randomly divided into a training set and a test set in the ratio of 8:2.

To validate the hydrolysis site prediction models, we collected hydrolysis data for 57 agrochemicals from Roberts’s Metabolic Pathways of Agrochemicals that were not used in the model. Additionally, hydrolysis data for 16 newly marketed agrochemical molecules reported in the literature between 2000 and 2024, along with two hydrolysis transformation examples utilized by Tebes-Stevens et al. [8], were gathered. Together, these datasets formed the external test set (Ext Data) for this study, comprising a total of 75 compound molecules. Detailed reaction examples for these compounds are provided in Table S2.

### 3.2. Atomic Vector Representation

The RDKit [30] cheminformatics software package was used to generate atomic environment fingerprints. Derived from the Morgan algorithm [31], these fingerprints effectively represent the local chemical environment of each atom within a molecule. The Morgan algorithm iteratively considers each atom and the chemical information of its neighboring atoms up to a predefined radius, which was set to 3 [32]. Each generated fingerprint is a 1024-bit binary vector, with each bit representing the presence or absence of a specific atomic environment or structural pattern within the molecule. Additionally, eight interpretable atomic descriptors were used to represent the chemical characteristics of the atoms, calculated by CDK [33]. The atomic descriptors selected and their meanings are listed in Table S3. By combining the atomic environment fingerprints and the atomic descriptors, a comprehensive feature set for each atom was constructed. These feature sets were used in the subsequent feature selection and model construction stages.

### 3.3. Feature Selection

Irrelevant, redundant, or noisy variable features will affect the accuracy and overall performance of any model; therefore, it is essential to employ feature selection prior to constructing a model using machine learning [34]. In this study, three feature selection methods were employed: variance threshold, ANOVA F-test, and *t*-test.

The variance threshold method was applied to exclude features that had the same value for all of the samples in the entire training set (i.e., features with zero variance). Then, the univariate ANOVA F-test was used to evaluate each feature, selecting the top 50% of features based on their F-values. Last, the *t*-test was introduced to assess the mean differences of each feature across different categories, retaining features with a *p*-value less than 0.05, indicating significant differences. Detailed instruments for these methods can be found at http://scikitlearn.org/stable/user_guide.html (accessed on 6 December 2024).

### 3.4. Model Building

#### 3.4.1. Model Pre-Training

During the model pre-training phase, preliminary training was conducted for each reaction type using eight machine learning algorithms. The models employed included light gradient boosting machine (LightGBM), random forest (RF), decision tree (DT), extra trees (ET), linear discriminant analysis (LDA), adaptive boosting (ADA), quadratic discriminant analysis (QDA), and gradient boosting classifier (GBC). Throughout the training process, 10-fold cross-validation was used to evaluate the performance of each model. The mean accuracy from the cross-validation on the training set was used as the performance metric to preliminarily select the models with better performance.

#### 3.4.2. Model Optimization

Based on the pre-training results, the five top-performing models for each reaction type were selected for further optimization. The hyperparameters of each model were fine-tuned using the “tune_model” function in PyCaret [35]. The performance of each set of hyperparameters was evaluated using 10-fold cross-validation. Finally, the optimal hyperparameter combination for each model was determined, and the accuracy improvements before and after the optimization were documented.

### 3.5. Model Validation

In the SOMs classification task, the performance of the classifiers was evaluated using several metrics: classification accuracy (ACC), recall, precision, and Matthews correlation coefficient (MCC). Ten-fold cross-validation was employed for these evaluations. The formulas for calculating these metrics are as follows:

ACC=(TP+TN)/(TP+FN+FP+FN)

Recall = TP/(TP + FN)

Pre = TP/(TP + FP)

$$MCC=\frac{TP\times TN-FP\times FN}{\sqrt{\left(TP+FP)(TP+FN)(TN+FP)(TN+FN\right)}}$$

TP, TN, FP, and FN represent the number of true positives, true negatives, false positives, and false negatives, respectively. The MCC is a metric that comprehensively considers the performance of binary classification models by taking into account the true positives, true negatives, false positives, and false negatives, providing more comprehensive information about the model’s performance.

### 3.6. Transformation Rules

#### 3.6.1. Rules Extraction

The transformation rules were extracted from Metabolic Pathways of Agrochemicals. This resource was meticulously analyzed to extract the hydrolysis reactions of various functional groups. The database includes the hydrolysis of carboxylic amides into carboxylic acids and amines, the conversion of nitriles to amides or carboxylic acids, the breakdown of esters into alcohols and acids, and similar transformations for carbonates, oximes, thioethers, and phosphothionate esters. These reactions were encoded into a computer-readable format to support accurate simulation in the prediction program. Each hydrolysis transformation was associated with specific environments, such as aquatic systems, plants, or animals, reflecting its relevance to these contexts.

#### 3.6.2. Conversion Rules to SMARTS Format

The extracted reaction rules were then converted into the SMARTS format, a line notation that specifies the substructures in molecules, making the rules computer readable. For example, the hydrolysis of a carboxylic amide was encoded as [#6:1] [C:2](=[O:3]) [N:4]>>([#6:1] [C:2](=[O:3]) [OH1]. [N:4]), representing the transformation of the carboxylic amide group into a carboxylic acid and an amine. A full list of the reaction types and their SMARTS can be found in Table S4.

#### 3.6.3. Rules Validation and Compilation

The known reactants and corresponding SMARTS strings were inputted into the program to generate the corresponding products, which were then compared with the actual known products to ensure rule accuracy. After the validation, the rules were compiled into the knowledge base, forming a robust set of hydrolysis transformation rules that the metabolic prediction program utilized for predicting chemical transformations.

### 3.7. Metabolite Prediction

Based on the defined transformation rules, the “Chemical Reactions” module in the RDKit cheminformatics software package was used to transform the parent molecules into possible metabolic products. The process was initiated by predicting the potential metabolic sites using metabolic site prediction models, which assign a probability score to each heavy atom in the molecule. Atoms with a probability score above a certain threshold were identified as potential sites for hydrolysis. Using these predictions, hydrolysis transformation rules were applied at the identified positions of the parent molecule to generate potential metabolites. For example, if an amide group was present at a predicted metabolic site, the corresponding hydrolysis reaction was simulated to produce a carboxylic acid and an amine. Each resulting metabolite was then scored based on the predicted probability of the metabolic site, with higher scores indicating a greater likelihood of formation.

## 4. Conclusions

Understanding the environmental fate of organic compounds is essential for understanding their behavior and impact within natural systems. Biotic and abiotic hydrolysis are critical processes that determine the stability and degradation of these compounds. This study integrated machine learning and expert systems to develop an efficient hydrolysis prediction model capable of accurately identifying hydrolysis sites and predicting hydrolysis products. The model leveraged atomic descriptors and atomic environment fingerprints, combining the intrinsic atomic properties with the surrounding environmental features to precisely identify the potential reaction sites. Within the model framework, the hydrolysis site prediction served as a critical preliminary step, prioritizing likely reactive sites to significantly reduce the search space and computational complexity associated with applying the reaction rules. This approach not only improved the predictive efficiency but also laid a solid foundation for the accurate matching of the reaction rules. Building on this foundation, the model incorporated a comprehensive library of reaction rules to simulate the hydrolysis process of the reactants and generate products with high precision, achieving reliable predictions of hydrolysis products.

The model, built using a limited sample dataset, demonstrated predictive performance comparable to the SyGMa model, which utilized three times the amount of sample data. Furthermore, the model successfully predicted 90% of the hydrolysis products in the external test set. These results underscore the robustness of the model and its potential applicability in predicting the hydrolytic transformations of diverse environmental organic compounds.

However, actual metabolic reactions are influenced by various complex processes and environmental factors. It may be beneficial to introduce more dimensions of data to further optimize the metabolic model. For instance, factors such as different pH levels, temperatures, and soil types can significantly impact hydrolytic metabolic reactions, as well as the interlinking mechanisms with other metabolic pathways. By considering these factors comprehensively, the model will be able to more accurately reflect the complexities of real-world applications, thus providing more scientific guidance for the metabolic processes of organic compounds.

## Figures and Tables

**Table 1:** Statistics information for the two datasets of SOMs.

	Sourcing Data		Ext Data	
Models	SOM+	SOM−	SOM+	SOM−
N-Hydrolysis	201	280	41	91
O-Hydrolysis	282	1025	68	163
C-Hydrolysis	40	1183	0	1169
Global-Hydrolysis	617	2576	113	1577

**Table 2:** Accuracy of the pre-trained model on the training set (10-fold cross-validation).

Reaction Types	N-Hydrolysis	O-Hydrolysis	C-Hydrolysis	Global-Hydrolysis
LightGBM	0.839	0.943	0.970	0.940
RF	0.839	0.946	0.978	0.948
DT	0.823	0.933	0.974	0.93
ET	0.836	0.944	0.972	0.947
LDA	0.841	0.934	0.968	0.910
ADA	0.818	0.936	0.971	0.909
QDA	0.804	0.875	0.972	0.871
GBC	0.813	0.935	0.973	0.926

**Table 3:** Comparison of the performance of different metabolite prediction tools.

	Our Model	GLORYx	SyGMa
Number of parent molecules	577	1438	1848
Total number of predictions	103	111	106
Number of true positives (out of 99)	90	97	90

## Data Availability

Data are contained within the article and Supplementary Materials.

## Supplementary Materials

The following supporting information can be downloaded at: https://www.mdpi.com/article/10.3390/molecules30020234/s1, Table S1: Model hyperparameter tuning; Table S2: Hydrolysis reactions in the Ext Set; Table S3: The atomic descriptors calculated by the CDK toolkit; Table S4: Reaction rules and reaction system. References [36,37,38,39,40,41,42,43,44,45,46,47,48,49,50] are cited in the Supplementary Materials.
